# Levofloxacin Prevention of Febrile Neutropenia after Cytarabine Consolidation in Acute Myeloid Leukemia

**DOI:** 10.31557/APJCP.2026.27.1.249

**Published:** 2026-01-21

**Authors:** Warangkana Assawawongsawat, Chayapa Thookhamme, Manassamon Navinpipat, Jakapat Vanichanan, Chantana Polprasert

**Affiliations:** 1 *Division of Hematology, Department of Medicine, Hatyai Hospital, Songkhla, Thailand.*; 2 *Department of Hematology, Chulabhorn Hospital, Chulabhorn Royal Academy, Bangkok, Thailand.*; 3 *Division of Infectious Diseases, Department of Medicine, Faculty of Medicine, Chulalongkorn University and King Chulalongkorn Memorial Hospital, Bangkok, Thailand.*; 4 *Division of Hematology, Department of Medicine, Chulalongkorn University and King Chulalongkorn Memorial Hospital, Bangkok, Thailand.*; 5 *Center of Excellence in Translational Hematology, Faculty of Medicine, Chulalongkorn University, Bangkok, Thailand. *

**Keywords:** Febrile neutropenia, Levofloxacin, acute myeloid leukemia, cytarabine consolidation

## Abstract

**Objective:**

This study aimed to assess the efficacy of Levofloxacin in preventing febrile neutropenia (FN) after cytarabine-based consolidation therapy in newly diagnosed acute myeloid leukemia (AML) patients.

**Methods:**

A prospective study conducted between January and December 2023. Newly diagnosed AML patients received Levofloxacin 750 mg once daily for 10 days, starting five days after the last cytarabine dose. Febrile neutropenia was diagnosed based on established guidelines, and outcomes were compared with a historical control group (January 2020 to December 2022) where Levofloxacin prophylaxis was not given.

**Results:**

A total of 43 patients were included, 55% were male with a median age of 42. Levofloxacin recipients (n=19) experienced significantly lower febrile neutropenia incidence compared to the control group (n=24) (36.8% vs. 100%, P<0.001). Levofloxacin prophylaxis also delayed the onset of febrile neutropenia (HR 0.14, 95% CI 0.06-0.36; P<0.001), reduced hospitalization rates (36.8% vs. 100%, P<0.01), and shortened hospital stays (4 vs. 7 days, P=0.002). There were no increases in antibiotic resistance or serious adverse events.

**Conclusion:**

Levofloxacin effectively reduced febrile neutropenia episodes, lowered hospitalization rates, and shortened hospital stays in post-cytarabine consolidation AML patients.

## Introduction

Acute myeloid leukemia (AML) is an aggressive hematologic malignancy. Without treatment, patients may die within 2-3 months [1]. Current treatment strategies aim to improve the survival rate, primarily through a combination of chemotherapy to induce disease remission followed by 3-4 cycles of consolidation therapy to eliminate remaining cancer cells [2, 3]. Intensive chemotherapy, however, usually causes a common side effect known as febrile neutropenia (FN) [4], which increases the risk of bloodstream infection and septicemia. 

The Thai Acute Leukemia Working Group conducted a study from January 2014 to December 2021, involving 992 AML patients. The findings revealed that individuals treated with intermediate-dose cytarabine (IDAC) experienced a high incidence of febrile neutropenia, reaching 72.9%. This event increased to 78.1% after treatment with high-dose cytarabine (HIDAC) during consolidation [5]. Patients also encountered a higher risk of severe infectious complications resulting in an elevated mortality rate [6].

In the context of infection prevention, guidelines from the National Comprehensive Cancer Network (NCCN) and the Infectious Diseases Society of America (IDSA) recommend the use of prophylactic antibiotics after chemotherapy in cancers, particularly fluoroquinolones, in patients with ANC less than 0.1x109/L for more than 7 days [6-10]. Due to concerns about the development of resistance to fluoroquinolone antibiotics [11, 12] and the increased incidence of *Clostridioides difficile*-related diseases [13, 14], the administration of antibacterial agents during periods of neutropenia has not yet become a standard practice.

Cytarabine, a pyrimidine analog, is widely used in the consolidation phase after achieving complete remission from the induction phase in AML. Pharmacodynamic studies on Cytarabine-induced neutropenia in AML patients were conducted through a retrospective cohort study involving 56 AML patients. The study found that neutrophil counts (ANC) began to drop below 1500 x 109/L approximately 7 days after administering Cytarabine and reached their lowest point between 10-14 days post-administration. Additionally, neutrophil levels decreased significantly faster in the HIDAC group compared to the IDAC group [15].

Levofloxacin is a broad-spectrum, bactericidal antibiotic in the fluoroquinolone drug class. It exhibits good tissue penetration, tolerability, and safety. It is commonly prescribed to patients with chemotherapy-induced neutropenia to prevent bacterial infections [16]. A prospective, multicenter, double-blind, randomized, placebo-controlled study in 2005 demonstrated that levofloxacin reduced the incidence of fever by 20% and lowered the rate of documented bloodstream infections by 16% in cancer patients (solid tumors, lymphoma, and leukemia) [17]. A meta-analysis and systematic review conducted in 2019 also supported the use of levofloxacin with acute leukemia patients (AML, ALL) following chemotherapy (induction, consolidation, or salvage chemotherapy), showing a significant reduction in the incidence of febrile neutropenia and lower infection rates compared to non-treated patients [18]. Lee SS, et al. conducted a retrospective single-center study at the London Health Science Center in 2018 revealing that levofloxacin reduced the hospitalization rate due to febrile neutropenia in AML patients after consolidation therapy (HIDAC, FLAG, or mitoxantrone combined with cytarabine) by 30%, without increasing the risk of *Clostridioides difficile*-associated disease [19].

However, a single-center retrospective study conducted by Vale CA, et al. in 2021 reported that administering antibacterial agents to AML patients after receiving IDAC/HIDAC did not reduce the incidence of febrile neutropenia. In contrast, the study found an increased risk of bloodstream infections and the presence of drug-resistant organisms, including fluoroquinolone-resistant gram-negative bacteria [20].

A review of previous studies shows there is no clear consensus on using antibacterial agents to prevent febrile neutropenia in AML patients receiving consolidation therapy. We aimed to explore the efficacy of levofloxacin administration in AML patients receiving cytarabine-based consolidation.

## Materials and Methods

### Study design

We conducted a prospective study compared to a historical control cohort at King Chulalongkorn Memorial Hospital, Bangkok, Thailand, from July 1, 2022, to March 31, 2024. Eligible participants included adults aged 18 to 65 with newly diagnosed acute myeloid leukemia post-cytarabine consolidation (IDAC/HIDAC). Ineligibility criteria included a history of fluoroquinolone allergy, QT prolongation on electrocardiography (males: QTc > 440ms, females: QTc > 460ms), use of other medications interacting with levofloxacin, use of other antibiotics for prophylaxis, receiving antibiotics within 5 days before enrollment, recent infection before enrollment, long-term fluoroquinolone use for another indication, or pregnancy.

In the prospective group, each patient received 750 mg of levofloxacin for ten days, starting on the fifth day after HIDAC or IDAC consolidation. Patients were scheduled for follow-up visits one and two weeks after chemotherapy to assess fever and monitor their complete blood count. Patients who developed a fever before a scheduled appointment were instructed to inform the research team and visit the hospital earlier for symptom assessment and blood tests. If febrile neutropenia was detected, immediate medical treatment was provided based on standard practice. The research team collected data on febrile neutropenia from the initiation of the first cycle of cytarabine consolidation until the final day of the last consolidation cycle or until the patient experienced febrile neutropenia. Informed consent for participation in the study was obtained in writing. The results were compared to those of a historical control group retrieved from medical records from January 2020 to December 2022, in which patients did not receive levofloxacin prophylaxis.

### Endpoints and assessment 

The primary endpoint was the incidence of clinically documented febrile neutropenia defined by a single body temperature measurement (oral/axillary) of ≥ 38.3 °C or a sustained body temperature of ≥ 38°C for 1 hour, and ANC < 500 cells/mm3 or an expected decrease of ANC to < 500 cells per mm3 in the next 48 hours [21]. Secondary endpoints included the rate of hospital admission, length of hospital stay due to febrile neutropenia, culture-positive bacteremia rate, composite outcome of septic shock rate and/or infection-related mortality, side effects of levofloxacin, incidence of *Clostridioides difficile*-associated disease, and fluoroquinolone resistance organisms.

### Statistical analyses

We estimated that a sample of 48 patients (24 patients per group) using a two-proportion test in a repeated-measure design would provide the trial with 80% power to detect a 30% reduction in the febrile neutropenia rate among AML patients undergoing fluoroquinolone post-consolidation chemotherapy compared to the no prophylaxis group. Intra-patient correlation was assumed at 0.5 with significance set at a two-sided alpha level of 0.05 [22-24]. 

Baseline characteristics were presented using appropriate descriptive statistics. Differences in the febrile neutropenia rate and the composite outcome of septic shock and/or infection-related mortality between groups were compared using Fisher’s exact test or the Chi-square test. The Kaplan–Meier method and log-rank test were used to analyze the time to the first febrile neutropenia event. Cox regression analysis was performed to calculate hazard ratios. The associations between clinical characteristics and febrile neutropenia were examined using both univariate and multivariable logistic regression analyses. A p-value of < 0.05 was considered statistically significant. The proportional hazards assumption was assessed using the Grambsch–Therneau test based on scaled Schoenfeld residuals.

## Results

A total of forty-three AML patients were included in the study. The median age of the patients was 42 years, and 55% were male. Among all patients, 54% had intermediate-risk AML, and the median cytarabine dose was 2.0 g/m². The historical comparison group without levofloxacin prophylaxis had a significantly higher BMI and BSA compared to the levofloxacin group. The nadir ANC post-cytarabine was not significantly different between the two groups, and G-CSF support was comparable between groups, with a median duration of 11 days in both cohorts, suggesting this variable did not influence the difference in febrile neutropenia incidence (Table 1). 

**Figure 1:**
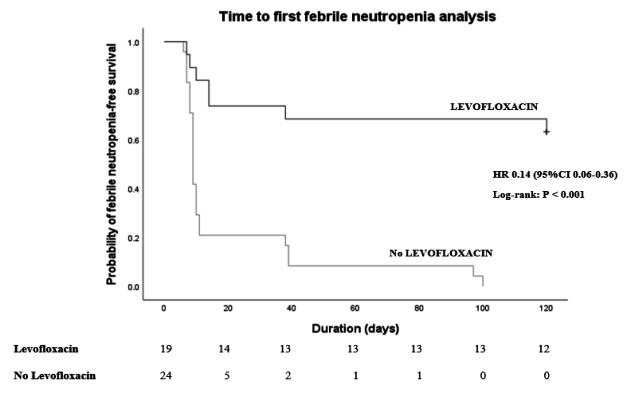
Kaplan-Meier Curve with Log-Rank Test Demonstrated Time to First Febrile Neutropenia Analysis. Comparison between levofloxacin and no levofloxacin prophylaxis groups by Cox regression analysis produced a hazard ratio with 95% confidence interval and P-value.

The percentage of patients with febrile neutropenia was lower in the levofloxacin arm compared to those without prophylaxis (36.8% vs. 100%, P<0.001). The majority of initial febrile neutropenia episodes occurred during the first cycle of cytarabine consolidation (Table 2). Levofloxacin prophylaxis also significantly reduced the time to the first febrile neutropenia episode compared to the group without levofloxacin (HR 0.14, P<0.001, 95% CI: 0.06–0.36), as shown in Figure 1. 

In the univariate analysis, higher BMI and BSA were significantly associated with an increased risk of febrile neutropenia (p = 0.01 and p = 0.03, respectively). However, these associations were not statistically significance in the multivariate model after adjusting for other variables (p = 0.19 and p = 0.65) (Table 3). For the proportional hazards assumption, the global test was not significant (Chi-square = 10.81, df = 14, p = 0.701), indicating no overall violation of the assumption. All individual covariates had p-values greater than 0.05, providing no strong evidence of non-proportional hazards (Supplementary Table S1). Levofloxacin prophylaxis significantly lowered the hospitalization rate (36.8% vs. 100%, P<0.01), and shortened length of stay (4 vs. 7 days, P=0.002). The composite outcome of septic shock and/or infection-related mortality was also lower, but not significant among the levofloxacin group (5.3% vs. 12.5%, P=0.62), (Table 4). The most common adverse event was nausea grade 1-2 (10.5%). No serious adverse events, including QT prolongation or cardiac toxicity, were observed during the study period. No increase in fluoroquinolone-resistant organisms or *Clostridioides difficile*-associated disease was reported (Table 5).

## Discussion

Results of this prospective study compared to historical control trials documented that the use of levofloxacin prophylaxis after cytarabine consolidation in newly diagnosed AML patients significantly reduced the incidence of febrile neutropenia. Our findings supported the established recommendations of the 2018 American Society of Clinical Oncology (ASCO) and the 2010 Infectious Diseases Society of America (IDSA) guidelines that recommended antibacterial prophylaxis with fluoroquinolones for high-risk patients with anticipated prolonged and profound neutropenia [6-8] in AML patients receiving consolidation therapy. 

Similar to our study, Weerapat et al. [18] and Lee et al. [19] noted a decrease in febrile neutropenia and related hospital admissions with levofloxacin prophylaxis. Of note, our study is the first to demonstrate a positive outcome specific to AML patients following cytarabine consolidation, which is currently a commonly used regimen.

In this study, Levofloxacin at a dose of 750 milligrams was chosen to increase the effectiveness of infection control, especially against *Pseudomonas aeruginosa,* which is commonly found in patients with neutropenia. Levofloxacin initiation on day 5 post-cytarabine was designed to align with the expected onset of neutropenia, typically occurring between days 7–14. This strategy was informed by prior pharmacodynamic studies and aimed to ensure prophylactic coverage during the period of highest infection risk.

Although patients in the levofloxacin group received a lower average dose of cytarabine compared to those in the non-levofloxacin group, reflecting evolving treatment recommendations that favor intermediate-dose cytarabine (IDAC) during post-remission therapy in AML [3], the nadir absolute neutrophil counts following cytarabine administration were not significantly different between the two groups. This suggests a comparable degree of neutropenia and supports the validity of comparing clinical outcomes between cohorts despite the difference in chemotherapy intensity. Nevertheless, the potential impact of cytarabine dosing on outcomes cannot be entirely excluded and remains a limitation of this study. 

Regarding the adverse effects of levofloxacin, this study did not find any serious adverse events or increases in antibiotic resistance. In contrast, Vale et al. [20] and De Rosa et al. [11] reported an elevated incidence of bacteremia and hospitalization due to drug-resistant organisms in the antibiotic prophylaxis group, with no difference in the febrile neutropenia rate. Notably, during that period, growth factor support was not administered with cytarabine consolidation, potentially contributing to prolonged neutropenia and increased infection risk. 

In our study, levofloxacin prophylaxis following cytarabine consolidation reduced the incidence of febrile neutropenia without apparent adverse effects. This practice should be encouraged in clinical settings, particularly for high-risk patients such as those receiving HiDAC, with higher BMI, older age, or multiple comorbidities who may derive the greatest benefit from levofloxacin prophylaxis.

The primary limitation of our study was the small sample size. Additionally, the relatively short follow-up period may have limited our ability to fully assess the potential long-term effects of levofloxacin, including the emergence of drug-resistant organisms or *Clostridioides difficile*-associated infections. Another limitation of this study is the use of a historical control cohort, which may introduce information bias. For example, earlier recognition of febrile episodes in the prospective group could have resulted from more direct communication with study staff.

In conclusion, levofloxacin prophylaxis demonstrated a significant benefit in preventing febrile neutropenia among AML patients undergoing cytarabine consolidation, with a favorable safety profile. A randomized controlled trial with a larger number of patients should be conducted to confirm this finding, which may provide strong evidence for a treatment recommendation in AML patients.

**Table 1:** Baseline Characteristics of the Patients in Both Groups

Characteristic	All (N=43)	Levofloxacin (N=19)	No Levofloxacin (N=24)	P-value*
Median age (year)	42 (15-63)	42 (19-63)	45 (15-63)	0.68
Age >= 60	6 (14.6%)	3 (15.8%)	3 (12.5%)	1
Male, n (%)	24 (55.8%)	8 (42.1%)	16 (66.7%)	0.25
ECOG	1 (1-3)	1 (1-2)	1 (1-3)	1
ECOG >2, n (%)	1 (2.3%)	0	1 (4.2%)	
Charlson Comorbidity Index	2 (2-8)	2 (2-6)	2.5 (2-8)	0.32
BMI <18.5 kg/m^2^, n (%)	6 (14%)	5 (26.3%)	1 (4.2%)	0.04
BMI 18.5-24.9 kg/m^2^, n (%)	28 (65.1%)	12 (63.2%)	16 (66.7%)	
BMI 25-30 kg/m^2^, n (%)	9 (20.9%)	2 (10.5%)	7 (29.2%)	
BSA (m^2^)	1.67 (1.34-2.10)	1.57 (1.34-1.85)	1.76 (1.38-2.10)	0.02
AML risk, n (%)				0.81
Favorable	15 (34.9%)	8 (42.1%)	7 (29.2%)	
Intermediate	23 (53.5%)	9 (47.4%)	14 (58.3%)	
Unfavorable	5 (11.6%)	2 (10.5%)	3 (12.5%)	
Median AraC dosage (g/m2)	2 (1.5-3)	1.5 (1.5-3)	3 (1.5-3)	0.92
IDAC, n (%)	19 (44.2%)	11 (57.9%)	8 (33.3%)	
HiDAC , n (%)	24 (55.8%)	8 (42.1%)	16 (66.7%)	
ANC before start CMT (x10^9^/L)	3822 (1620-10610)	4186 (1710-10610)	3085 (1620-7830)	0.8
Nadir ANC post-AraC (x10^9^/L)	42 (0-310)	56 (0-310)	30 (0-250)	0.39
Duration of GCSF (day)	11 (5-17)	11 (5-17)	11 (6-15)	0.33

BMI, Body mass index; BSA, Body surface area; AraC, Cytarabine; IDAC, intermediate-dose cytarabine; HIDAC, high-dose cytarabine; ANC, Absolute neutrophil count; CMT, chemotherapy. *Chi-square and Independent T test or Mann-Whitney test

**Table 2:** Incidence of Febrile Neutropenia in Both Groups

Characteristic	Levofloxacin (N=19)	No Levofloxacin (N=24)	P-value*
Febrile neutropenia, n (%)	7 (36.8%)	24 (100%)	<0.001
Cycle of 1st febrile neutropenia, n (%)			
1stcycle	4 (21.05%)	19 (79.17%)	
2ndcycle	2 (10.53%)	3 (12.5%)	
3rdcycle	0 (0%)	0 (0%)	
4th cycle	1 (5.26%)	2 (8.33%)	

*Fisher’s exact test

**Table 3:** Factors Associated with Febrile Neutropenia

Characteristic	Univariate analysis		Multivariate analysis	
	OR (95%CI)	P-value	OR (95%CI)	P-value
Age ≥ 60	0.74 (0.12-4.7)	0.75		
Sex (Male)	2.22 (0.57-8.60)	0.25		
ECOG > 2	NA	1		
CCI ≥3	2.47 (0.56-10.92)	0.23	1.96 (0.36-10.61)	0.44
BMI	1.47 (1.09-1.98)	0.01	1.35 (0.87-2.10)	0.19
BSA	103 (1.65-6435.04)	0.03	4.60 (0.01-3004.04)	0.65
AML risk				
Intermediate	1.42 (0.34-5.87)	0.63		
Unfavorable	2.00 (0.17-22.95)	0.58		
Dose AraC (> 1.5 g/m^2^)	1.39 (0.36-5.28)	0.63		
Duration of G-CSF (> 7 days)	0.22 (0.03-1.98)	0.18	0.28 (0.03-3.00)	0.29

CCI, Charlson Comorbidity Index; BMI, Body mass index; BSA, Body surface area; Arac, Cytarabine.

**Table 4:** Secondary Outcomes of Both Groups

Characteristic	Levofloxacin (N=19)	No Levofloxacin (N=24)	P-value
Composite outcome of septic shock and/or infection-related mortality, n (%)	1 (5.3%)	3 (12.5%)	0.62
Septic shock, n (%)	1 (5.3%)	3 (12.5%)	
Infection-related mortality, n (%)	0	0	
Hospitalization, n (%)	7 (36.8%)	24 (100%)	<0.01
Length of stay (days)	4 (0-20)	7 (4-14)	0.002
Culture-positive bacteremia, n (%)	1 (5.3%)	3 (12.5%)	0.62
Gram-negative organism, n (%)	1 (5.3%)	2 (8.3%)	
Gram-positive organism, n (%)	0	1 (4.2%)	

**Table 5:** Summary of Adverse Events in Patients Receiving Levofloxacin Prophylaxis

Toxicity of Levofloxacin (N=19)	
Side effect, n (%)	2 (10.5%)
*Clostridioides difficile *-associated disease, n (%)	0 (0%)
Fluoroquinolone resistance organism, n (%)	0 (0%)

## Author Contribution Statement

Warangkana Assawawongsawat performed material preparation, data collection and analysis. Chayapa Thookhamme, Manassamon Navinpipat, Jakapat Vanichanan and Chantana Polprasert provided clinical information. Jakapat Vanichanan and Chantana Polprasert conceptualized and designed the study. The first draft of the manuscript was written by Warangkana Assawawongsawat and all authors commented on previous versions of the manuscript. All authors read and approved the final manuscript. 

## References

[1] Döhner H, Weisdorf DJ, Bloomfield CD (2015). Acute myeloid leukemia. N Engl J Med..

[2] Shukla n, walter rb (2022). American society of hematology self-assessment program [internet].

[3] Döhner H, Wei AH, Appelbaum FR, Craddock C, DiNardo CD, Dombret H (2022). Diagnosis and management of aml in adults: 2022 recommendations from an international expert panel on behalf of the eln. Blood..

[4] Gupta A, Singh M, Singh H, Kumar L, Sharma A, Bakhshi S (2010). Infections in acute myeloid leukemia: An analysis of 382 febrile episodes. Med Oncol..

[5] Chanswangphuwana C, Polprasert C, Owattanapanich W, Kungwankiattichai S, Rattarittamrong E, Rattanathammethee T (2022). Comparison of three doses of cytarabine consolidation for intermediate- and adverse-risk acute myeloid leukemia: Real world evidence from thai acute myeloid leukemia registry. Clin Lymphoma Myeloma Leuk..

[6] Freifeld AG, Bow EJ, Sepkowitz KA, Boeckh MJ, Ito JI, Mullen CA (2011). Clinical practice guideline for the use of antimicrobial agents in neutropenic patients with cancer: 2010 update by the infectious diseases society of america. Clin Infect Dis..

[7] National comprehensive cancer network (2020). Prevention and treatment of cancer-related infections (version 2. https://www.nccn.org.

[8] Hwang S, Kwon KT, Kim Y, Bae S, Chang HH, Kim SW (2021). Usefulness analysis of the 2018 asco/idsa guideline for outpatient management of fever and neutropenia in adults treated for malignancy. Sci Rep..

[9] Imran H, Tleyjeh IM, Arndt CA, Baddour LM, Erwin PJ, Tsigrelis C (2008). Fluoroquinolone prophylaxis in patients with neutropenia: A meta-analysis of randomized placebo-controlled trials. Eur J Clin Microbiol Infect Dis..

[10] Kusick K, Earl MA, Gallagher EM, Yeh JY, Advani AS, Kalaycio ME (2011). Fluoroquinolone prophylaxis in acute myeloid leukemia (aml) patients undergoing post-remission chemotherapy reduces hospital admission rates. Blood..

[11] De Rosa FG, Motta I, Audisio E, Frairia C, Busca A, Di Perri G (2013). Epidemiology of bloodstream infections in patients with acute myeloid leukemia undergoing levofloxacin prophylaxis. BMC Infect Dis..

[12] Kern WV, Klose K, Jellen-Ritter AS, Oethinger M, Bohnert J, Kern P (2005). Fluoroquinolone resistance of escherichia coli at a cancer center: Epidemiologic evolution and effects of discontinuing prophylactic fluoroquinolone use in neutropenic patients with leukemia. Eur J Clin Microbiol Infect Dis..

[13] McCusker ME, Harris AD, Perencevich E, Roghmann MC (2003). Fluoroquinolone use and clostridium difficile-associated diarrhea. Emerg Infect Dis..

[14] Johnson S, Lavergne V, Skinner AM, Gonzales-Luna AJ, Garey KW, Kelly CP (2021). Clinical practice guideline by the Infectious Diseases Society of America and Society for Healthcare Epidemiology of America: 2021 focused update on management of Clostridioides difficile infection in adults. Clin Infect Dis..

[15] Shepshelovich D, Edel Y, Goldvaser H, Dujovny T, Wolach O, Raanani P (2015). Pharmacodynamics of cytarabine induced leucopenia: A retrospective cohort study. Br J Clin Pharmacol..

[16] Podder v Levofloxacin. Statpearls [internet].

[17] Bucaneve G, Micozzi A, Menichetti F, Martino P, Dionisi MS, Martinelli G (2005). Levofloxacin to prevent bacterial infection in patients with cancer and neutropenia. N Engl J Med..

[18] Owattanapanich W, Chayakulkeeree M (2019). Efficacy of levofloxacin as an antibacterial prophylaxis for acute leukemia patients receiving intensive chemotherapy: A systematic review and meta-analysis. Hematology..

[19] Lee SSF, Fulford AE, Quinn MA, Seabrook J, Rajakumar I (2018). Levofloxacin for febrile neutropenia prophylaxis in acute myeloid leukemia patients associated with reduction in hospital admissions. Support Care Cancer..

[20] Vale CA, Egan PC, Ingham R, Farmakiotis D, Reagan JL (2021). Antibiotic use during cytarabine consolidation in acute myeloid leukemia. Ann Hematol..

[21] Hughes WT, Armstrong D, Bodey GP, Bow EJ, Brown AE, Calandra T (2002). 2002 guidelines for the use of antimicrobial agents in neutropenic patients with cancer. Clin Infect Dis..

[22] Brown h, prescott r (2006). Applied mixed models in medicine.

[23] Diggle p, liang ky, zeger s (1994). Analysis of longitudinal data.

[24] Liu H, Wu T (2005). Sample size calculation and power analysis of time-averaged difference. J Mod Appl Stat Methods..

