# Supplementary material for: Levofloxacin Prevention of Febrile Neutropenia after Cytarabine Consolidation in Acute Myeloid Leukemia
Source: Asian Pac J Cancer Prev. 2026 Jan 21;27(1):249–54. doi: 10.31557/APJCP.2026.27.1.249 (PMC13418041; doi:10.31557/APJCP.2026.27.1.249)
Supplement: Table S1 [file APJCP-27-1-249-s001.pdf]

Supplementary Table S1:

Proportional Hazards Assumption Checks Using Schoenfeld Residuals

Characteristic	Chi-square	df	P-value
Age (year)	3.10	1	0.08
Sex	0.42	1	0.52
ECOG>2	0.39	1	0.53
CCI	1.78	4	0.77
BMI	0.05	1	0.63
BSA	0.60	1	0.44
AML risk	1.72	2	0.42
Dose AraC (g/m2)	0.23	1	0.63
Actual dose AraC (gm)	0.89	1	0.34
Levofloxacin	0.12	1	0.73
**GLOBAL**	10.81	14	0.701
